# Expedient Access to Gold/Quantum‐Dot Nanohybrids Mediated by Poly(ethylene Glycol) Ligands of Distinct Macromolecular Architecture

**DOI:** 10.1002/marc.202500657

**Published:** 2025-11-02

**Authors:** Olga V. Kuharenko, Artsiom Antanovich, Avijit Saha, Aliaksei Ivanchanka, Martin Müller, Vladimir Lesnyak, Annette Kraegeloh, Christian Rossner

**Affiliations:** ^1^ Leibniz‐Institut für Polymerforschung Dresden e.V. Dresden Germany; ^2^ Faculty of Chemistry and Food Chemistry Technische Universität Dresden Dresden Germany; ^3^ Physical Chemistry Technische Universität Dresden Dresden Germany; ^4^ Institute of Physical Chemistry and Electrochemistry Leibniz University Hannover Hannover Germany; ^5^ INM‐Leibniz Institute for New Materials Saarbrücken Germany; ^6^ Department of Polymers University of Chemistry and Technology Prague Prague Czech Republic

**Keywords:** block‐copolymers, core−satellite nanostructures, gold nanoparticles, hybrid nanomaterials, non‐covalent interactions, poly(ethylene glycol), quantum dots

## Abstract

We report a straightforward methodology to access structurally well‐defined hybrid assemblies of plasmonic and excitonic nanoparticles (NPs). The developed strategy is based on the incorporation of quantum dots (QDs) coated with zinc‐sulfide shells into poly(ethylene glycol) (PEG) brushes at gold NP surfaces, without the necessity of incorporating specialized functional groups to drive the supracolloidal assembly. Based on control experiments involving PEGs with distinct polymeric architecture and Fourier‐transform infrared spectroscopy analysis, we attribute the structure formation to attractive interactions between the QD surface and the monomeric repeat unit of the PEG brushes. This combination leads to short interparticle spacings and plasmon/exciton interactions, resulting in photoluminescence (PL) quenching upon assembly. However, using block‐copolymers comprising a NP‐adjacent spacer block in addition to a NP‐remote PEG block, the distance between gold NPs and QDs can be controlled, which in turn affects the PL properties. The versatility of the structure‐formation approach is demonstrated by the possibility of applying it to two distinct core/shell QDs (InP/ZnSe/ZnS and CdSe/CdS/ZnS). This offers new perspectives in the quest for efficient nanomaterial fabrication procedures.

## Graphical abstract

We demonstrate that distinct quantum dots with ZnS shells can be incorporated directly into poly(ethylene glycol) brushes at gold nanoparticle surfaces, based on non‐covalent interactions between the quantum‐dot surface and PEG monomer. The structure and photoluminescence properties of obtained gold/quantum‐dot conjugates can be engineered by adjusting the macromolecular design of the poly(ethylene glycol) ligands.

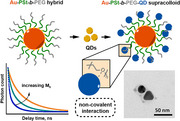

## 1 Introduction

Supracolloidal assemblies that combine plasmonic and excitonic NPs are promising for a range of applications, including plasmon‐enhanced (photo‐)catalysis [1, 2], light‐emitting devices [3, 4], and (bio‐)sensing [5, 6]. These applications are enabled by plasmon‐exciton coupling interactions, which depending on the distance between the plasmonic and excitonic NPs can result in luminescence quenching or enhancement [7]. This has been demonstrated for layered structures comprising a gold‐NP monolayer on a glass surface, a thickness‐defined separating layer prepared by layer‐by‐layer deposition of polyelectrolytes, covered by a top layer comprising QDs [8, 9]. Later, similar behavior was observed in colloidal solutions of core−satellite‐type supracolloids [10, 11], comprising plasmonic NP cores and QD satellite particles, separated by polyelectrolyte layers [12], a dielectric (SiO_2_ or Al_2_O_3_) layer of variable thickness [13, 14], or DNA linkers [15]. Such core−satellite‐type assemblies are especially promising due to their well‐defined arrangement structure. This has allowed the development of several sensing strategies, wherein in vitro detection of biomolecular targets is achieved by linking entities containing a specific receptor. An example is given by luminescence‐quenched gold/QD conjugates, in which a specific enzyme activity cleaves the conjugates with concomitant restoration of the QD luminescence, enabling enzyme‐activity assays [5, 16] (e.g. for beta‐site Amyloid precursor protein‐cleaving enzyme 1, involved in the proteolytic formation of Amyloid‐beta peptides, which accumulate during the pathogenesis of Alzheimer's disease) [5]. In a similar manner, specific target single‐stranded DNA can be detected by gold/QD nanohybrids linked via complementary base‐pairing [17]. Remote sensing of physical parameters such as local temperature [18] around plasmonic NPs becomes possible with synthetic polymer linkers that process temperature information by changes in their swelling degree, such as in nanoscale thermometers in which poly(ethylene glycol) (PEG) linkers connect gold NPs and QDs [19].

Several synthetic approaches have been developed to fabricate such well‐defined core−satellite‐type hybrid assemblies of plasmonic and excitonic NPs. One of the strategies relies on linking by biomacromolecular species, such as hybridization between constituent NPs that were functionalized with complementary single‐stranded DNA [15, 20], assembly mediated by rigid DNA origami bundles [21], biotin/streptavidin binding [22], or linking by bovine serum albumin [23]. Alternatively one can employ fully synthetic macromolecular species, for example (i) adsorption of QDs onto (polystyrene‐*block*‐poly(4‐vinyl pyridine)) block‐copolymer shells physisorbed on AuNPs [24], (ii) incorporation of QDs into micelles composed of ABA‐type (poly(acrylic acid)‐*block*‐polystyrene‐*block*‐poly(acrylic acid)) triblock‐copolymers, where the outer A block forms the QD‐containing micellar core and AuNPs are attached to trithiocarbonate groups in the center of the B‐type middle blocks, which form loops protruding from the micellar core [25], and (iii) encapsulation of QDs by a cross‐linked poly(isoprene)‐*block*‐poly(ethylene glycol) (PI‐*b*‐PEG) block‐copolymer matrix chemically modified with lipoic acid for gold binding [26], or (iv) embedding QDs in PI‐*b*‐PEG micelles covered with an additional polystyrene shell, which provides a defined 5–15 nm spacing to attached AuNPs [27]. While powerful, these approaches are based on specific interactions between the NPs and the organic linking entities, and thus require complex multi‐step procedures. Thus, it is highly desirable to facilitate synthetic access to structurally well‐defined plasmonic/excitonic nanohybrids, ideally employing readily available components in a straightforward manner.

**SCHEME 1:**
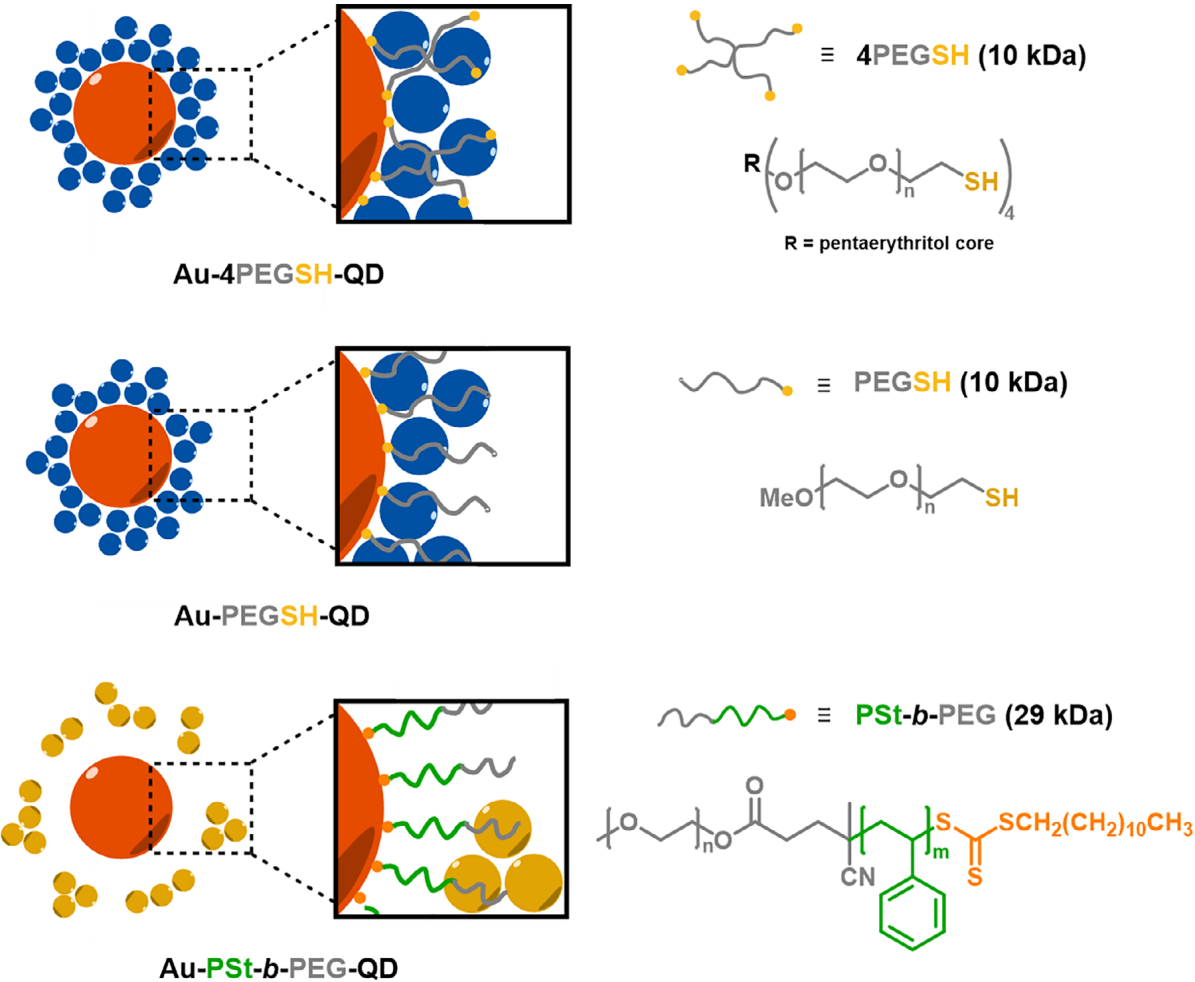
Schematic overview of the gold/QD supracolloids presented in the current study.

Our work reports a simplified and generalizable strategy toward the assembly of gold NP/QD core–satellite supracolloids utilizing solely PEGs with terminal sulfur‐containing groups as soft linkers. The fabrication method combines well‐established Au–S anchoring with previously underexplored non‐covalent coordination between PEG ether oxygens and QD surfaces. As a result, we achieve uniform and stable supracolloids without arduous pre‐functionalization, demonstrating that PEG linkers can drive assembly through intrinsic chemical affinity. Moreover, we show that engineering the polymeric architecture of the PEG‐containing linker component provides positional control over QD satellites in the gold/QD conjugates and directly impacts their PL behavior. Thereby, our strategy offers a simple, versatile, and tunable platform for constructing well‐defined hybrid gold/QD nanomaterials (Scheme 1).

## 2 Results and Discussion

**FIGURE 1:**
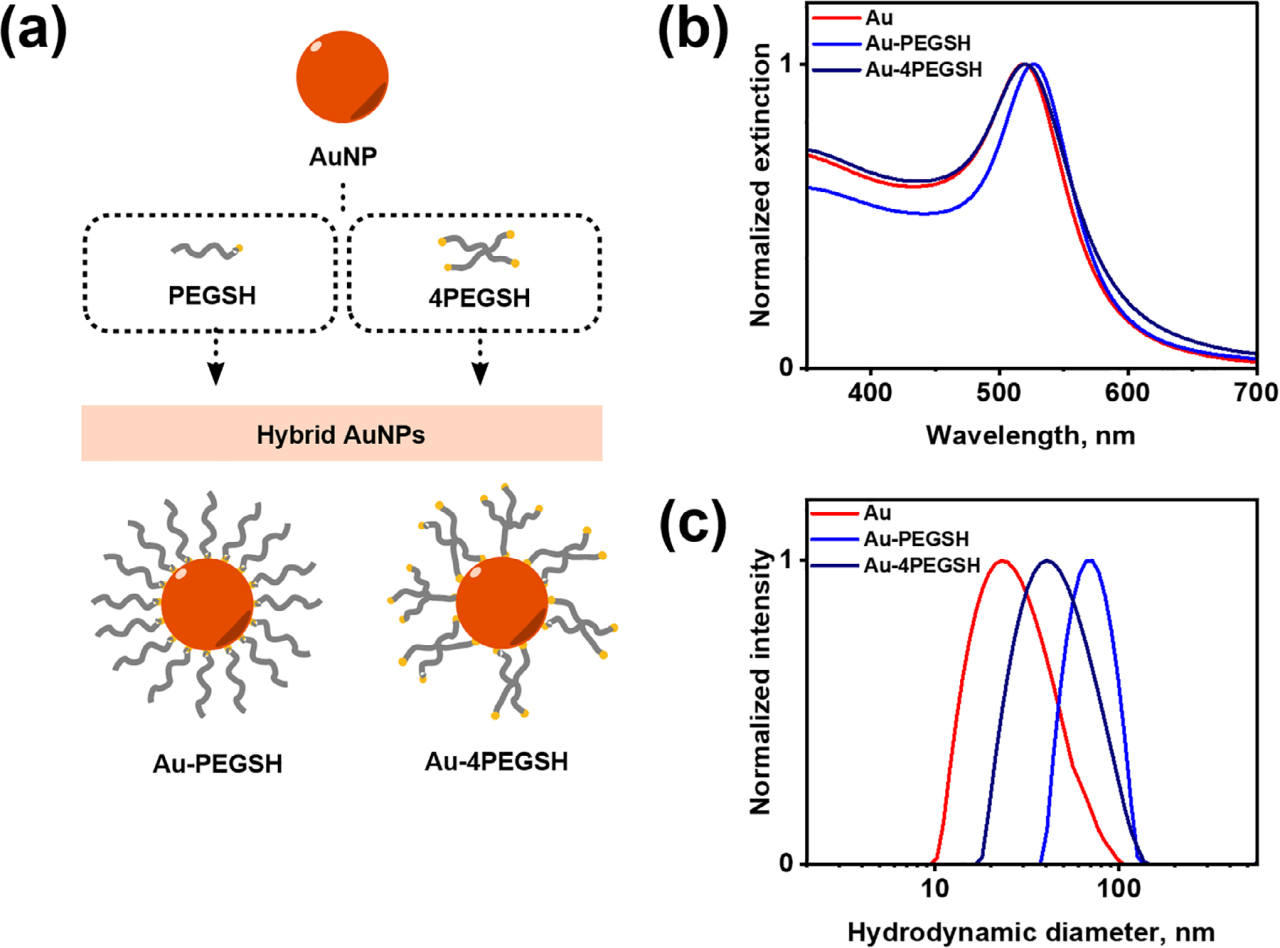
(a) Schematic representation of AuNP functionalization using linear PEGSH and 4‐arm star‐shaped 4PEGSH ligands; (b) extinction spectra of 15 nm AuNPs and derived nanohybrids (15Au‐PEG) in water; (c) DLS size‐distribution curves of 15 nm AuNPs and 15Au‐PEG nanohybrids in water.

PEG‐coated gold NPs were synthesized from quasi‐spherical citrate‐capped gold NPs (15 and 30 nm, Figure S1) by adsorption of polymer ligands in a grafting‐to approach [28, 29]. PEG ligands with linear (PEGSH) and star topology (4‐arm PEGSH, or 4PEGSH), providing thiol end groups on one chain or the star branch termini, respectively, were employed (Figure 1a). Successful surface modification was proven by visible extinction spectroscopy. For citrate‐stabilized 15 nm AuNPs the localized surface plasmon resonance (LSPR) maximum was observed at 519 nm, while after PEGSH and 4PEGSH functionalization the peaks shifted to 527 and 520 nm, respectively (Figure 1b; Figure S2a). The redshift in the case of linear PEGSH reflects effective replacement of the citrate shell with PEG ligands and the associated increase in the local refractive index at the NP surface [30]. In contrast, functionalization with branched 4PEGSH does not induce a pronounced redshift but instead leads to a slight broadening of the extinction spectrum between 550 and 700 nm, which we attribute to the onset of weak interparticle interactions or partial aggregation upon grafting with the star‐shaped macromolecules.

Complementary information was obtained from dynamic light scattering (DLS) for both Au‐PEGSH (∼70 nm) and Au‐4PEGSH (∼45 nm) nanohybrids compared to the original citrate‐stabilized AuNPs (Figure 1c; Figure S2b). Such values are expected, as DLS captures the inorganic core together with the hydrated polymer corona and associated ion/solvent layers, and the intensity‐weighted analysis emphasizes contributions from even a small fraction of larger scatterers. The smaller hydrodynamic size of Au‐4PEGSH compared to Au‐PEGSH is consistent with the more compact architecture of the star polymer. For a star‐shaped PEG, the overall molar mass is distributed across four arms, which shortens the effective arm length and thus reduces the corona thickness. In contrast, linear PEGSH chains extend further into solution, producing a thicker shell and larger apparent size.

**FIGURE 2:**
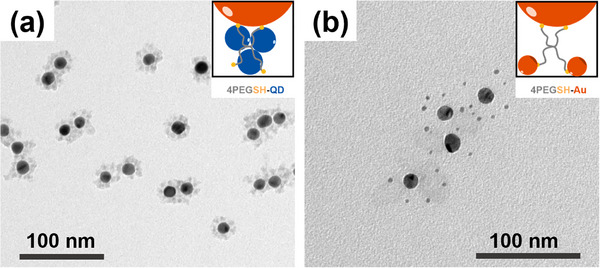
TEM images of (a) 15Au‐4PEGSH‐CdSe/CdS/ZnS and (b) 15Au‐4PEGSH‐Au supracolloids dried from DMF.

We started our investigations into the formation of hybrid gold/QD supracolloids from 15 nm gold NPs coated with 4‐arm PEGSH ligands. To ensure solvent compatibility with colloidal solutions of CdSe‐ or InP‐based QDs dispersed in *N*‐methylformamide (NMF), the Au‐4PEGSH core−shell particles were transferred from water to *N,N*‐dimethylformamide (DMF) and then introduced into QDs solution to initiate assembly. Transmission electron microscopy (TEM) analysis of the assembly structures revealed AuNPs surrounded by multiple QD satellites, which can be distinguished from AuNPs due to their distinct size and contrast, with a defined satellite arrangement structure and short interparticle spacings (Figure 2a). The assembly yield was quantified by UV/vis extinction spectroscopy and found to be consistently high (≥70%), see Figure S3 for details. For comparison, we also prepared supracolloids using small (4 nm) AuNPs instead of QDs as satellites in otherwise similar assembly conditions. Here, considerably larger interparticle distance between the gold core and gold satellites was found (Figure 2b). These findings are consistent with earlier results [31] that established the binding of gold satellites through free‐, non‐adsorbed gold‐affine star polymer termini in the polymer shell. The separation of these free end groups from the gold core is determined by the degree of polymerization of the star polymer linker. Since the gold satellites are captured by these free end groups during the assembly process, their distance from the gold core roughly coincides with the distribution of these end groups (see inset in Figure 2b) [31]. The fact that CdSe/CdS/ZnS QDs on the other hand penetrate into the PEG brush points to other specific interactions that drive the supracolloidal assembly. A pure size effect can be excluded, since the employed QD satellites are larger than the gold satellites (Figure S4), which does not promote their penetration into the brush layer [32]. At the same time, it should be emphasized that TEM probes dried samples where soft polymer coronas such as PEG collapse and may obscure the actual situation in dispersion. Therefore, only semi‐quantitative conclusions about distinct interparticle distances observed by TEM can be drawn.

**FIGURE 3:**
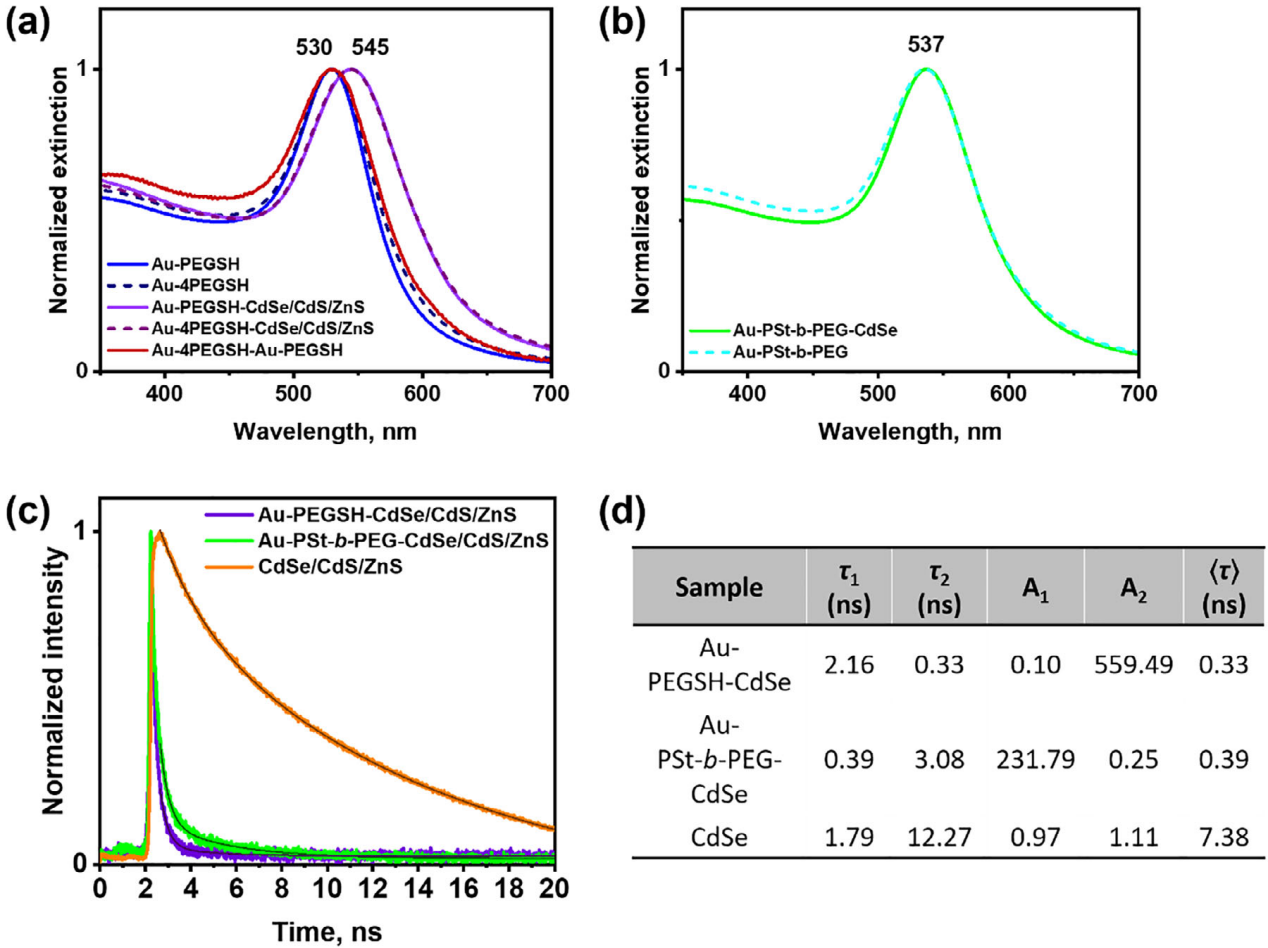
(a) Extinction spectra of 30Au‐PEG nanohybrids, 30Au–Au, 30Au‐PEG‐CdSe/CdS/ZnS nanoassemblies and (b) 30Au‐PSt‐*b*‐PEG nanohybrids, and 30Au‐PSt‐*b*‐PEG‐CdSe/CdS/ZnS nanoassemblies in DMF, (c) PL life‐time decay curves of CdSe/CdS/ZnS QDs and 30Au–CdSe/CdS/ZnS nanoassemblies in DMF and (d) time‐resolved PL decay parameters of 30Au‐PEGSH‐CdSe/CdS/ZnS, 30Au‐PSt‐*b*‐PEG‐CdSe/CdS/ZnS, and CdSe/CdS/ZnS.

The structural differences between the two types of assemblies (Au–CdSe/CdS/ZnS and Au–Au) are reflected in the optical properties of the supracolloids, which we explored for 30 nm gold core NPs (Figure 3a). Extinction spectra of the Au–CdSe/CdS/ZnS supracolloids exhibit LSPR centered at 545 nm, compared to 530 nm for the resonance position of Au‐PEG NPs. This redshift can be attributed to an altered dielectric environment around the Au core affected by the presence of many QDs close to the gold surface [33]. On the other hand, coupling interactions with the gold satellites are rather weak due to the large interparticle spacing (and the comparably small size of the satellite particles), resulting in only minor changes to the extinction spectrum in the case of gold−gold supracolloids (Figure 3a).

**FIGURE 4:**
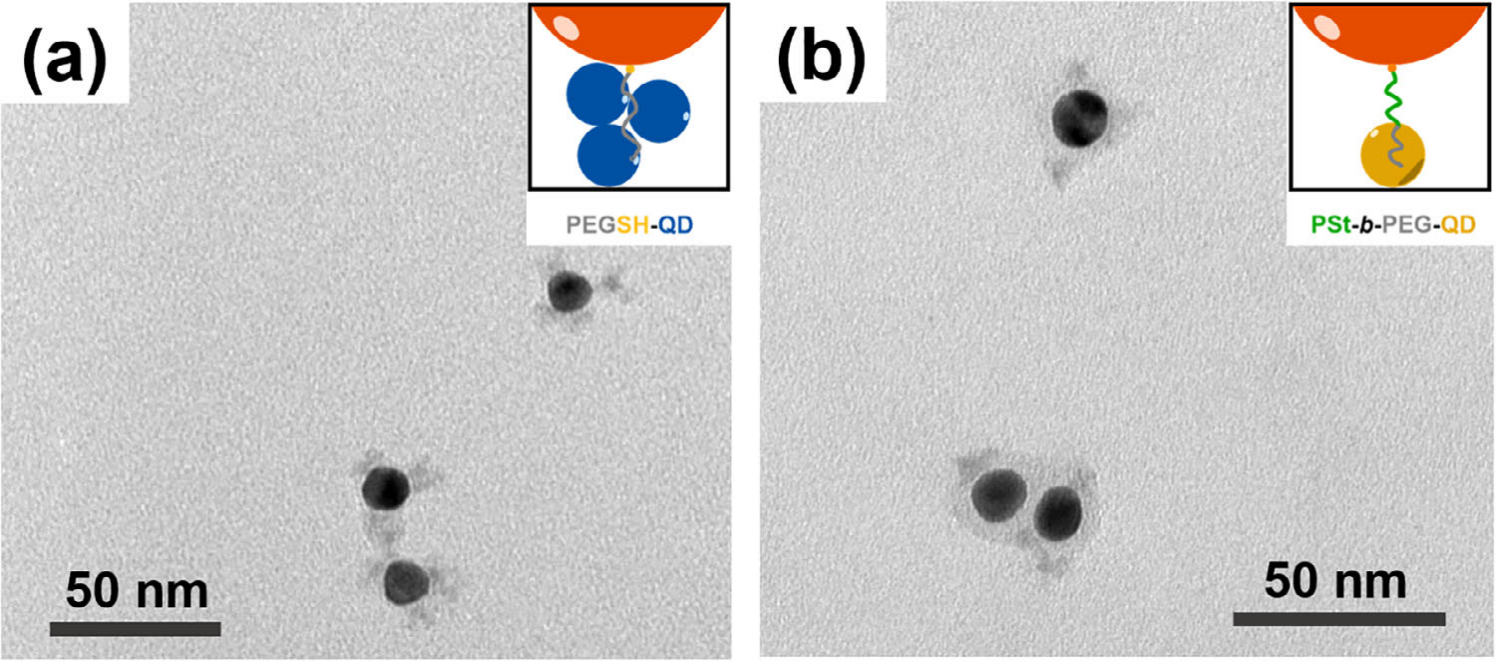
TEM images of (a) 15Au‐PEGSH‐CdSe/CdS/ZnS and (b) 15Au‐PSt‐*b*‐PEG‐CdSe/CdS/ZnS supracolloids dried from DMF.

In the next step, we modified the architecture of the PEG ligand. Firstly, monofunctional linear PEGSH was used instead of four‐arm star‐shaped PEG, which also resulted in the formation of supracolloidal structures as confirmed by TEM (Figure 4a; Figure S5) and extinction spectroscopy (Figure 3a). The presence of a comparable LSPR shift (to 545 nm) both in Au‐4PEGSH‐CdSe/CdS/ZnS and Au‐PEGSH‐CdSe/CdS/ZnS systems indicates that QD–PEG interactions are sufficiently strong to drive assembly even when thiol groups are absent in the PEG brush layer. PL measurements further reveal that the emission from CdSe/CdS/ZnS QDs in the nanoassemblies is almost completely quenched (Figure S6a,b). A similar optical behavior is observed in Au–InP/ZnSe/ZnS assemblies, where the PL of InP/ZnSe/ZnS QDs is fully quenched (Figure S6c,d), which is consistent with a short spatial separation between the Au core and the QD satellites.

In an attempt to tune the gold/QD spacing and potentially mitigate PL quenching observed for homo‐PEG‐linked Au/QD supracolloids, we prepared PSt‐*b*‐PEG block‐copolymer by reversible addition−fragmentation chain transfer (RAFT) polymerization as an alternative linker, where the PSt block acts as a spacer between (gold‐binding) trithiocarbonate groups and the PEG block (Figure S7). Extinction spectroscopy measurements demonstrated AuNPs functionalized with RAFT PSt‐*b*‐PEG exhibited a stronger redshifted LSPR (to a peak maximum at 537 nm) compared to those modified with thiolated PEGs (530 nm), indicating differences in local dielectric environment, due to the inner PSt block. Notably, no further redshift was observed upon QD binding to Au‐PSt‐*b*‐PEG hybrids, which is in contrast to the behavior observed for QD incorporation into homo‐PEG layers at AuNPs, where the LSPR position is strongly affected by QD incorporation (Figure 3b).

To assess the effect of the PSt spacer on optical interaction between Au cores and QDs, time‐resolved photoluminescence (TRPL) measurements were performed (Figure 3c). The average lifetimes, obtained by two‐exponential fits are summarized in Figure 3d. Compared with the Au‐PEGSH‐CdSe/CdS/ZnS assemblies, Au‐PSt‐*b*‐PEG‐CdSe/CdS/ZnS exhibited a ∼15% slower PL decay, which reflects the distinct positioning of the QDs with regard to AuNP. In Au‐PEGSH‐based clusters, QDs are located in close proximity to the Au surface, resulting in rapid non‐radiative energy transfer, while in Au‐PSt‐*b*‐PEG‐based clusters, the PSt block acts as a spacer, partially preserving a fraction of the intrinsic exciton lifetime. Consistent behavior was observed in both DMF and chloroform (Figure S8a). As good solvents for PSt, these media favor extended polymer conformations, which help maintain the increased Au–QD separation and ensure reproducible improvements in PL decay times across different dispersions. Spacing between the gold core and QDs is corroborated by TEM imaging: QDs were found to interact selectively with the outer PEG segment, while no QDs were incorporated into the inner PSt block, which thus effectively acted as a spacer that increased the overall interparticle distance (Figure 4b; Figure S8b).

**FIGURE 5:**
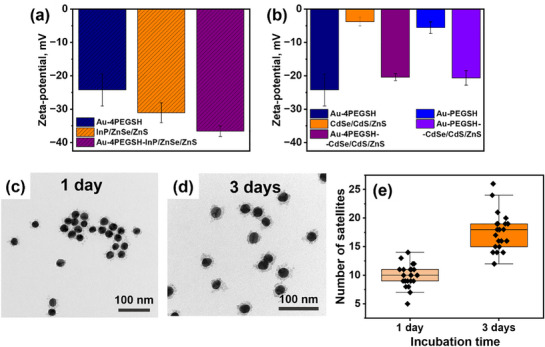
Zeta‐potential of (a) 30Au‐4PEGSH, InP/ZnSe/ZnS QDs, and 30Au‐4PEGSH‐ InP/ZnSe/ZnS in DMF; (b) 30Au‐4PEGSH, CdSe/CdS/ZnS, 30Au‐4PEGSH‐CdSe/CdS/ZnS, 30Au‐PEGSH, and 30Au‐PEGSH‐CdSe/CdS/ZnS in DMF. TEM images of 30Au‐4PEGSH‐CdSe/CdS/ZnS obtained after (c) 1 day and (d) after 3 days of incubating 30Au‐4PEGSH nanohybrids with CdSe/CdS/ZnS QDs. (e) Distribution of CdSe/CdS/ZnS satellites per Au core after 1 and 3 days of incubation based on the TEM images analysis.

To assess potential electrostatic effects on supracolloidal assembly, we conducted electrophoretic (zeta‐potential) measurements. These reveal negative zeta‐potential values for the gold/PEG particles (which can be attributed to remaining citrate not completely removed during grafting of PEG ligands) [34] and as well for the InP‐ and CdSe‐based QDs (Figure 5a,b). Thus, supracolloidal assembly proceeds regardless of repulsive electrostatic interactions between the two types of colloidal particle building blocks. However, compared with InP/ZnSe/ZnS QDs, the zeta‐potential of CdSe/CdS/ZnS QDs used in this study is close to neutral (Figure 5b), minimizing repulsive forces and facilitating the approach of QDs to the PEG‐coated Au surface. The presence of like surface charges on both the gold NP cores and the QD satellites is reflected in the rate of the supracolloidal assembly, which proceeds on the time scale of several hours up to few days: TEM images taken after 1 day of assembly show moderate amount of QD satellites surrounding the gold NP core (Figure 5c,e). However, after 3 days, the same system exhibits a significantly higher number of CdSe/CdS/ZnS satellites per core (Figure 5d,e). This comparably slow assembly kinetics mirrors the free‐energy barriers involved in the assembly of like‐charged colloidal species.

**FIGURE 6:**
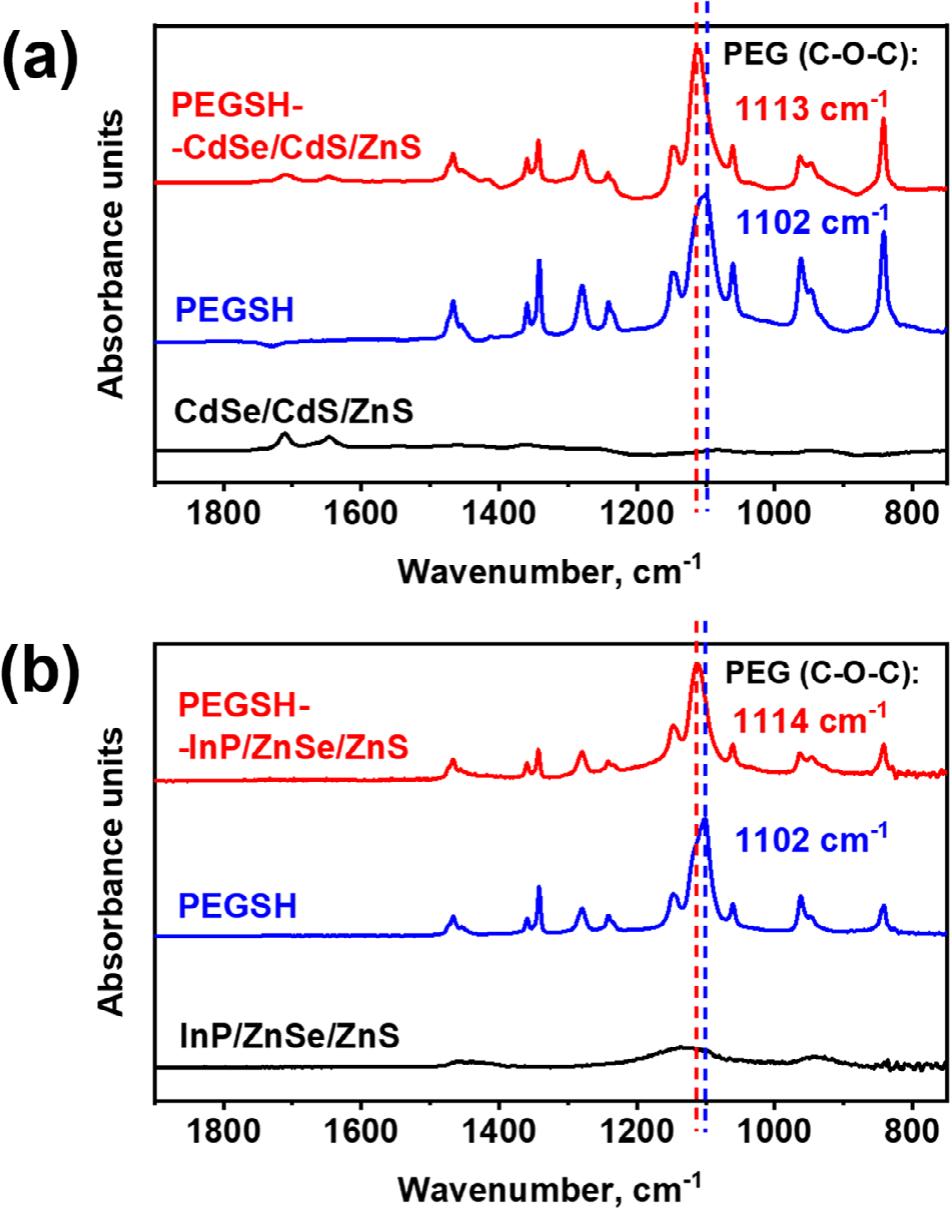
(a) FTIR spectra of CdSe/CdS/ZnS QDs, PEGSH and PEGSH‐CdSe/CdS/ZnS hybrids. (b) FTIR spectra of InP/ZnSe/ZnS QDs, PEGSH and PEGSH‐InP/ZnSe/ZnS hybrids.

To summarize the findings at this stage: The binding of QDs to PEG‐grafted AuNPs is not driven by thiol groups in the PEG brush, as demonstrated by control experiments; the QDs are selectively incorporated into the PEG brush, but not into a PSt spacer block; and QD uptake into the PEG brush proceeds despite repulsive electrostatic interactions between the constituent gold and QD colloids. From these observations, it may be concluded that considerably strong specific interactions between the PEG monomer and the QD surface drive the assembly process. To shed light onto these interactions, FTIR experiments have been performed (Figure 6a,b).

Upon binding of PEGSH to CdSe‐based QDs, a clear blueshift of the characteristic ether stretching band (ν(C─O─C)) was observed—from 1102 cm^−1^ in free PEGSH to 1113 cm^−1^ in PEGSH‐CdSe/CdS/ZnS complex (Figure 6a). A similar shift was observed between free PEGSH (1102 cm^−1^) and PEGSH bound to InP‐based QDs, where the ν(C─O─C) band of the latter appeared at 1114 cm^−1^ (Figure 6b), suggesting identical binding behavior in both systems. Such spectral shifts between the free and bound state of PEG systems are well known and related assignments are given in the following.

At first, according to Bailey [35] and Rozenberg [36], lower wavenumber positions of the antisymmetric ν(C─O─C) band at around 1104 cm^−1^ can be assigned to trans torsional (“anti”) conformers with respect to R‐O‐CH_2_‐CH_2_‐O‐R moieties of PEG, indicating rather stretched conformations. Higher wavenumber positions at around 1127 cm^−1^ can be assigned to respective gauche conformers, indicating rather coiled conformations. Secondly, Rozenberg [36] reported that with increasing water content of PEG solutions the lower “trans component” at 1104 cm^−1^ is progressively increasing, while the higher “gauche component” at 1127 cm^−1^ is decreasing. Hence, the observed blueshift of the ν(C─O─C) oscillator is likely attributed to changes in both conformation and solvation taking into account that atmospheric water readily interacts with hydrophilic PEG compounds.

Accordingly, before QD interaction, free and well‐solvated PEG chains at the AuNP surface prevail in a rather extended state and thus the ν(C─O─C) oscillator is softened (red shift). Whereas, after QD interaction PEG chains wrap tightly around the included QDs upon specific coordination interaction between the PEG oxygens (lone pairs) and—most likely—Zn centers or hydrogen‐bond donors from mercaptopropionic acid ligands. Upon this process proximal bound H_2_O molecules can be liberated from the PEG moieties, rather coiled conformations formed, and thus the ν(C─O─C) oscillator stiffened (blue shift).

Hence, FTIR spectroscopy could indicate rather “side‐on” interactions between QDs (CdSe/CdS/ZnS, InP/CdS/ZnS) and PEGSH via EG segments (excluding rather “end‐on” interactions). This finding suggests similar specific interactions within hybrid systems composed of satellite QD and PEG brush‐modified planet AuNP.

## 3 Conclusions

We developed a strategy for fabricating Au−QD core−satellite nanoclusters using PEGs of distinct polymeric architecture as macromolecular surface ligands that mediate the assembly process. PEG‐based macromolecules form brushes on gold NP surfaces and capture QDs into the polymer brush. The colloidal interactions involved in this novel assembly mechanism were identified in control experiments employing PEGs with distinct macromolecular design and by means of FTIR spectroscopy, indicating non‐covalent interactions between the QD surface and the PEG side chain. After establishing the interaction of the QD surface with PEG monomers we can give the following explanation for the short separation of QDs from the gold surface within supracolloids assembled using homo‐PEGs: When polymer‐molecule ligands are grafted to a convex surface, in a good solvent the monomer density decreases monotonously with increasing distance from the surface in the semi‐dilute brush regime, as has been demonstrated based on scaling arguments [37, 38] and corroborated by molecular dynamics simulations [39] and small‐angle X‐ray scattering [40]. Thus, to maximize interactions with the PEG repeat unit, the QDs locate close to the gold core where the monomer density is high. A comparable behavior was recently observed in the co‐assembly of PEG‐grafted gold NPs with other gold NPs carrying polymer ligands with hydrogen‐bond donor moieties along the main chain to drive the particle assembly: After supplying energy (by sonication), the supracolloids approached arrangement structures with very short particle spacings [41]. Due to the short interparticle spacings, coupling interactions between the constituent NPs are maximized, which is desirable for applications that benefit from plasmon/exciton coupling. While in the systems based on homo‐PEG ligands presented herein short interparticle spacings led to fast luminescence decay, increased luminescence lifetimes are achieved by introducing a spacer block in PSt‐*b*‐PEG block‐copolymer ligands that increased the core–satellite distance. This demonstrates how the structure and PL properties of gold/QD conjugates can be engineered by targeted macromolecular design of the PEG‐based linker components. Exploring the scope of this original supracolloidal assembly strategy especially in terms of the polymeric architecture is subject to further work and may provide new prospects for (bio)sensing strategies.

## Conflicts of Interest

The authors declare no conflicts of interest.

## Supporting information




**Supporting File**: marc70104‐sup‐0001‐SuppMat.docx.

## Data Availability

The data supporting this article have been included as part of the Supporting Information. Specific data files (such as instrument files or data spreadsheets) related to this study are available upon request from the corresponding author.
