# Supplementary material for: Expedient Access to Gold/Quantum‐Dot Nanohybrids Mediated by Poly(ethylene Glycol) Ligands of Distinct Macromolecular Architecture
Source: Macromol Rapid Commun. 2025 Nov 2;47(1):e00657. doi: 10.1002/marc.202500657 (PMC12784180; doi:10.1002/marc.202500657)
Supplement: Supplementary file 1 — Supporting File: marc70104‐sup‐0001‐SuppMat.docx. [file MARC-47-e00657-s001.docx]

Supporting Information

Expedient access to gold/quantum-dot nanohybrids mediated by poly(ethylene glycol) ligands of distinct macromolecular architecture

Olga V. Kuharenko, Artsiom Antanovich, Avijit Saha, Aliaksei Ivanchanka, Martin Müller, Vladimir Lesnyak, Annette Kraegeloh, Christian Rossner*

O. V. Kuharenko, A. Ivanchanka, M. Müller, C. Rossner

Leibniz-Institut für Polymerforschung Dresden e.V., D-01069 Dresden, Germany.
E-mail: rossner@ipfdd.de

O. V. Kuharenko, C. Rossner

Faculty of Chemistry and Food Chemistry, Technische Universität Dresden, D-01069 Dresden, Germany.

A. Antanovich, A. Saha, V. Lesnyak
Physical Chemistry, Technische Universität Dresden, 01069 Dresden, Germany.

A. Antanovich
Institute of Physical Chemistry and Electrochemistry, Leibniz University Hannover, Callinstraße 3a, D-30167 Hannover, Germany.

A. Kraegeloh

INM-Leibniz Institute for New Materials, Campus D2 2, Saarbrücken 66123, Germany.

C. Rossner

Department of Polymers, University of Chemistry and Technology Prague, Technická 5, Prague 6 16628, Czech Republic.

**Materials and Methods**

*Materials.*

Hydrogen tetrachloroaurate(III) trihydrate (≥99.9%, HAuCl_4_·3H_2_O), sodium citrate tribasic dihydrate (≥99%), O-(2-Mercaptoethyl)-O´-methyl-polyethylene glycol (PEGSH, 10 kDa), poly(ethylene glycol) methyl ether (4-cyano-4-pentanoate dodecyl trithiocarbonate) (PEG CTA, 5.4 kDa), styrene (St), indium(III) chloride (99.99%, InCl_3_), oleyl amine (70%, OlAm), 1-octadecene (90%, ODE), tri-octylphosphine (97%, TOP), tris(diethylamino)phosphine (97%, (DEA)_3_P), zinc(II) chloride (99.999% trace metals basis), zinc stearate (10-12% Zn basis, Zn(St)_2_), zinc acetate (99.99%), 1-dodecanethiol (≥98%, DDT), tetrabutylammonium hexafluorophosphate (99%), sulfur powder (99.999%), mercaptopropionic acid (≥99%, MPA), *N*-methylformamide (99%, NMF), ammonium bromide (≥ 99.99% ), ammonium iodide (99.999%), cadmium oxide (99.5%, trace metals basis), n-hexane (≥ 95%), myristic acid (98.5%), selenium powder (160 mesh, 99.99%), thiourea (95%), triethylene glycol dimethyl ether (99%), toluene (≥ 99.8%), zinc oxide (≥ 99.0%), dichloromethane (≥99.5%, DCM) and chloroform (≥99.8%, CHCl_3_) were purchased from Sigma Aldrich and used as received. Isopropanol (99.8%), and oleic acid (OlAc, technical grade, 90%) were purchased from Fisher Chemical. Zinc bromide (98%) and zinc iodide (98%) were purchased from Alfa Aesar. 4-arm PEGSH (10 kDa, 4PEGSH) was purchased from JenKemTechnology and used as received. *N,N*-dimethylformamide (≥99%, DMF) was purchased from Acros Organics and used as received. Milli-Q water was used in all experiments. All glassware for preparation of nanoparticles was cleaned with aqua regia and rinsed extensively with Milli-Q water before use.

*Synthesis of gold nanoparticles.*

Synthesis of 15 nm spherical core gold nanoparticles:^S1^ A hot solution of sodium citrate in MilliQ-water (5 ml, 39 mM, 3.9 equiv.) in a 250 mL three-neck round-bottom flask was injected to the boiling solution of HAuCl_4_ (100 ml, 0.5 mM, 1.0 equiv.) under vigorous stirring. The mixture was refluxed for 15 min and cooled down to room temperature. Results from TEM characterization are given in Figure S1 a and b.

Synthesis of 30 nm spherical core gold nanoparticles:^S1^ A solution of sodium citrate in MilliQ-water (2.2 mM, 150 mL) in a 250 mL three-neck round-bottom flask was heated in an oil bath to boiling and refluxed for 15 min. Then, 1 mL of aqueous HAuCl_4_ (25 mM) was injected. In 10 min the color of the solution changed from yellow to bluish gray and then to pale-pink. The reaction mixture was immediately cooled down in the same vessel to 90 °C and 1 mL of sodium citrate (60 mM) and 1 mL of HAuCl_4_ (25 mM) were sequentially injected with a time delay of 2 min. After 30 min, the reaction was cooled down at room temperature. Results from TEM characterization are given in Figure S1 c and d.

Synthesis of 4 nm satellite gold nanoparticles:^S2^ In a 100 ml round-bottom flask, a solution of HAuCl_4_ (0.0945 g, 0.240 mM) in MilliQ-water (20 mL) and tetra-*N*-octyl ammonium bromide (0.5249 g, 0.960 mM) in toluene (20 ml) were mixed and stirred until the aqueous layer had turned colorless. The aqueous layer was removed using a syringe. To the remaining organic layer, sodium borohydride (0.0726 g, 1.92 mM) in MilliQ-water (5 mL) was added dropwise under vigorous stirring, that lead to the color change from wine-red to an intense ruby red. The reaction mixture was stirred for further 5 h. The organic phase was separated, sequentially washed with dilute sulfuric acid (5 %, 10 mL) and water (5 × 10 mL) and dried over anhydrous magnesium sulfate.

*Synthesis of InP/ZnSe/ZnS QDs.*

Se-precursor (2M): 1.578 g of Se-pellets were dissolved in 10 mL of TOP at room temperature by constant stirring overnight in a nitrogen-filled glove box.

S-precursor (2M): 0.64 g of S-powder was dissolved in 10 mL of TOP at room temperature by constant stirring in the glovebox.

QD synthesis: InP QDs were synthesized using a hot-injection method based on the previously reported approach^S3^ with minor modification. In a typical synthesis, 100 mg (0.45 mmol) of InCl_3_ and 300 mg (2.20 mmol) of ZnCl_2_ and 3 mL (9.10 mmol) of OlAm were loaded into a round-bottom (RB) three-necked flask attached to a Schlenk line. The reaction mixture was degassed at 120 °C under a vacuum with constant stirring. The phosphorus precursor was prepared inside a glove box by dissolving 0.55 mL (2 mmol) of (DEA)_3_P in 1 ml of OlAm. This solution was heated to 100-120 °C accompanied by evaporation of diethylamine. After 1 hour of degassing, the temperature of the RB flask containing indium precursor was quickly (35-40 °C min^−1^) increased to 220 °C in an Ar atmosphere. As soon as the temperature reached 220 °C the (DEA)_3_P precursor was swiftly injected into the reaction mixture. The reaction temperature was maintained for 30 min for the growth of the InP QDs. The reaction was terminated by removing the heating mantle and the colloidal solution cooled down to room temperature.

The growth of ZnSe/ZnS shell was performed by some modification of a literature method.^S4^ 120 mg of tetrabutylammonium hexafluorophosphate, 2 g of Zn(St)_2_ powder, 0.2 mL of water, 3 mL of ODE and 2 mL of OlAm were mixed with the as-synthesized InP QDs in a three-necked RB flask coupled to a Schlenk line and degassed at 120 °C under vacuum. After 1 h of degassing the temperature of the reaction flask was set to 300 °C in an Ar atmosphere. During the temperature ramp, 1.6 mL of a 2 M TOP-Se solution was injected at 150 °C. The reaction temperature (300 °C) was maintained for 40 min for the growth of the ZnSe shell. Subsequently, the temperature of the reaction flask was reduced to 100 °C.

For the ZnS shell growth, 400 mg of zinc acetate was added to the reaction mixture and further degassing was carried out for 1 h. Subsequently, 1 mL of a 2M TOP-S solution was injected at 150 °C in Ar atmosphere while raising the temperature to 330 °C. The reaction temperature was maintained for 1 h for the ZnS shell growth. Finally, the QDs solution was cooled down to room temperature using a water bath. QDs were purified 3-4 times using a toluene/ethanol mixture (1:4 vol.), centrifuged at 7000 rpm for 5 min, followed by dispersion of the obtained precipitate in toluene and stored for further use.

Ligand Exchange: For the biphasic ligand exchange, InP/ZnSe/ZnS QDs were first precipitated by the addition of ethanol followed by centrifugation. The precipitate was dried under argon flow and redissolved in octane at a concentration of 15 mg mL^-1^. A solution of ZnI₂ was prepared by dissolving 478 mg of ZnI₂ in 6 mL of NMF using a vortex mixer. Subsequently, 3 mL of this ZnI₂ solution was transferred to a vial containing an additional 3 mL of NMF and 300 μL of MPA. 6 mL of QDs in octane were added to the solution to form a biphasic mixture. This mixture was vigorously stirred for 1-2 h until the QDs transferred to the polar phase. The bottom layer, containing the QDs, was collected and purified twice via centrifugation using a toluene/hexane mixture. The obtained precipitate was dried under argon flow and dissolved in NMF using the vortex mixer.

*Synthesis of CdSe/CdS/ZnS QDs.*

QD synthesis: 128 mg (1 mmol) of CdO, 685 mg (3 mmol) of myristic acid, and 15 mL of ODE were degassed in a 100 mL three-neck flask for 5 min. The mixture was the heated to 270 °C under Ar until the complete CdO dissolution and cooled to 90 °C, degassed for 1h and then flushed with argon. In a separate flask, 39 mg (0.5 mmol) of Se was dispersed in 1 mL of ODE, the mixture was bubbled with Ar and transferred into a syringe. The selenium suspension was quickly injected into a cadmium myristate solution heated to 260 °C, and the flask was kept at 250 °C for 5 min for the QD growth. Afterwards, the temperature was decreased to 180 °C and 300 μL of 0.1 M solution of sulfur in ODE was slowly added dropwise. Then, the heating mantle was removed, 1 mL of OlAc was added into the reaction mixture and the flask was allowed to cool to 90 °C. The QDs were isolated by adding excess isopropanol with following centrifugation.

The precipitate was redissolved in 10 mL of ODE and transferred into a 100 ml three-neck flask containing zinc 2-ethylhexanoate solution obtained by heating 40 mg (0.5 mmol) of ZnO in 240 μL (1.5 mmol) of 2-ethylhexanoic acid and 1 mL of ODE. The flask was heated to 90 °C, degassed for 1 h, and flushed with Ar. In a separate flask, 120 mg (1.5 mmol) of ZnO were dissolved in 4.5 mL of 2-ethylhexanoic acid under heating until the solution became transparent. Separately, 76 mg (1 mmol) of thiourea were dissolved in 1.5 mL of triethylene glycol dimethyl ether. After both solutions cooled down to room temperature, they were mixed, bubbled with Ar for 10 min and then transferred into a syringe. The ZnS growth solution was slowly added dropwise into the CdSe QD solution over 30 min at 180 °C. After the addition was complete, the flask was kept at 180 °C for 15 minutes. Then the heating mantle was removed, and the flask was allowed to cool down to 80 °C. The QDs were isolated by adding excess isopropanol with following centrifugation. The precipitate was redissolved in chloroform and cleaned through two more precipitation/dissolution cycles using chloroform and isopropanol as a solvent and a non-solvent, respectively.

Ligand exchange: 700 μL of the solution of CdSe/CdS/ZnS QDs in chloroform (75 mg mL^−1^) were mixed with ~500 μL of isopropanol and centrifuged. Obtained QD pellet was dissolved in 3 mL of hexane, and then 3 mL of NMF was added to form a bilayered mixture. 1 mL of 0.25 M ZnI_2_ and 0.5 M NH_4_I was added to the biphasic solution, which was vigorously shaken until QD phase transfer was completed. To facilitate the separation of the two phases, 500 μL of toluene were added. After the separation, the transparent and colorless hexane layer was discarded, and a 1:1 chloroform/toluene mixture was added until the solution became turbid. Afterwards, the solution was centrifuged, the precipitate was dissolved in a fresh portion of neat NMF (slight sonication to facilitate dissolution might be needed), and the precipitation procedure was repeated one more time.

*Synthesis of PSt-b-PEG copolymer*

Prior to use, styrene monomer was purified from the stabilizer by Al_2_O_3_ basic column.

A mixture of PEG CTA (80.5 mg, 0.0149 mmol) and styrene (428 μL, 3.73 mmol) was degassed with Ar for 30 min and heated at 100 °C for 18h. The resulting polymer was purified via precipitation from THF to cold hexane for four times and dried in the vacuum (M_n_^GPC^ = 29 kDa, M_w_/M_n_ = 1.03).

*Colloidal assembly of core-satellite Au–QD nanostructures.*

In a typical experiment 30 nm AuNPs cores were concentrated from 1 ml of initial colloidal solution by centrifugation (8000 rcf, 10 min) and redispersed in aqueous solution of 10 kDa PEGSH (1 ml, 0.5 mg mL^−1^). After overnight incubation, PEG-coated AuNPs were subjected to four sequential centrifugation/redispersion cycles in water (4500 rcf, 15 min) to remove excess polymer. See Figure S2 a and b for characterization by extinction spectroscopy and DLS, respectively.

Prior to modification with QDs, Au-PEG nanohybrids were concentrated by centrifugation (4500 rcf, 15 min) and redispersed in 300 μL DMF. To 300 μL Au-PEG in DMF, 10 μL CdSe- or InP-based QDs in NMF (*c*(QDs)=20 mg mL^−1^) were added under stirring and left in the fridge for 1 or 3 days respectively. After incubation Au-PEG-QD nanoclusters were purified from the excess of QDs via three centrifugation/redispersion cycles in DMF (3000 rcf, 15 min). In order to transfer Au-PEG-QD nanoclusters into an aqueous medium, one more additional centrifugation step (3000 rcf, 15 min) was performed followed by redispersion in water.

Based on the literature data,^S6^ PEGSH grafting densities on AuNPs are expected to follow a size-dependent trend, with saturation densities of ~0.82 chains/nm^2^ for 16.2 nm AuNPs and ~0.69 chains/nm^2^ for 28.0 nm AuNPs. Extrapolating from these results, the grafting density on 30 nm AuNPs used in this work can be expected fo fall in a similar range (0.6-0.7 chains/nm^2^), reflecting the reduced curvature of larger cores.

*Yield determination of core-satellite Au–QD nanostructures.*

The assembly yield of Au–QD supracolloids was quantified by UV/vis extinction spectroscopy with respect to the AuNP concentration. Gold concentration was determined from the extinction at 400 nm, where the interband transition provides a shape- and size-independent extinction coefficient.^S7^ Extinction spectra were first measured for Au-polymer nanohybrids, followed by the measurement of the corresponding Au-polymer-QD assemblies, enabling direct comparison of Au concentrations before and after QD addition. Based on this approach, the yield of 15Au-PEGSH-CdSe assemblies was calculated to be ~81%, while 15Au-PSt-*b*-PEG-CdSe assemblies showed a yield of ~73% (see Figure S3). Yields that deviate from quantitative conversion are attributed to unavoidable material loss during sample handling (e.g., decantation to remove excess QDs), rather than to intrinsic differences in the assembly efficiency: TEM analysis of the assemblies (Figure 2a, Figure S4b, and Figure S5a) confirms that after QD addition essentially all gold cores are surrounded by QD satellites with no significant population of unassembled Au-polymer particles remaining, validating this optical method for yield estimation.

We note that QDs also absorb in the 400 nm region, however, their extinction coefficient is more than an order of magnitude lower than that of 15 nm AuNPs.^S8,S9^ Even for assemblies containing up to ~10 QDs per AuNP, the QD contribution to the extinction at 400 nm is negligible to a good approximation (˂5-10%) compared to gold, and therefore does not strongly affect the yield determination.

*Formation of core-satellite Au–Au nanostructures.*

The assembly procedure was performed according to previous work.^S5^ A solution of 4 nm AuNPs (0.5 mL, in toluene) was added to Au-4PEGSH nanohybrids dispersed in 0.5 mL DCM at room temperature and the mixture was shaken for 30 min. After that, a solution of linear PEGSH in DCM (0.5 mL, 5 mg mL^−1^) was added and the mixture was shaken for 30 minutes and then centrifuged for 20 min at 4500 rcf. The material was redispersed in 1 mL DCM and then again centrifuged for 20 min at 4500 rcf, and finally redispersed in 1 mL of DMF.

*Characterization.*

Transmission electron microscopy (TEM) was performed using Zeiss TEM Libra 120 at an accelerating voltage of 120 kV.

Dynamic light scattering (DLS) was performed using Anton Paar Litesizer 500 with a laser wavelength of 658 nm. Zeta-potential measurements were performed using the same instrument equipped with a reusable cuvette (Univette, Anton Paar GmbH).

UV-Vis extinction spectroscopy was performed with a spectrometer Cary 5000 (Agilent Technologies Deutschland GmbH). For gold-containing nanomaterials the extinction at 400 nm was used to determine the concentration.^S7^

Photoluminescence spectra were recorded using a FluoroMax-4 spectrofluorometer (Horiba Jobin Yvon Inc.).

Time resolved photoluminescence measurements were carried out on PicoQuant Instruments (MicroTime 200, PicoQuant, Germany) by utilizing an inverted optical microscope (100X, air objective) in solution phase using the time-correlated single-photon counting technique (TCSPC). The experiments were performed with a single-photon counting module (SPCM-AQRH, Excelitas, USA) by using a 405 nm picosecond diode laser (LDH-D-C-405, PicoQuant, germany) at a repetition rate of 40 MHz. The lifetime was calculated by fitting the intensity decay with the two-exponential tail-fit.

ATR-FTIR measurements were performed on a Vertex 70 FTIR spectrometer (Bruker Optics GmbH, Ettlingen, Germany) equipped with ATR-FTIR four-mirror setup (Perkin Elmer, Switzerland), globar source and DTGS detector. ATR-FTIR spectra were recorded collecting 25 scans at spectral resolution of 2 cm^-1^ (zero filling factor 2). OPUS software (Bruker Optics GmbH, Ettlingen, Germany) was used for spectral evaluation. Trapezoidal internal reflection elements (IRE) made of germanium (Ge, 50 x 20 x 2 mm^3^) (KOMLAS GmbH, Berlin, Germany) were treated by plasma cleaning (PDC 32G, Harrick, Ossining, US) under reduced pressure and then located in an in-situ ATR-FTIR cell (M. Müller, IPF Dresden) sealed by O-rings. At first reference intensity spectra I_R_ of the bare Ge IRE surface were recorded. Then 10-50 microliters of the polymer/quantum dot (P/QD) suspensions in CHCl_3_ were spread onto the surface of the Ge IRE and sample intensity spectra I_S_ recorded under nitrogen flow to eliminate residual CHCl_3_ and loosely bound water in the P/QD deposit. ATR-FTIR spectra of P/QD deposits (A) were obtained by A = -log(I_S_/I_R_).

Gel-permeation chromatography (GPC) was performed using Agilent + Wyatt Technology modular system consisting of a high-performance liquid chromatography pump 1100, an 1100 ALS autosampler and two PLgel-MIXED-C columns (300×7.5 mm^2^, Agilent Technologies, US) equipped with a refractive index (Optilab T-rEX from Wyatt Technology, US) and light scattering (DAWN HELEOS from Wyatt Technology, US) detectors. As an eluent, THF with 0.025% butylhydroxytoluene at a flow rate of 1ml min^-1^ was used.

^1^H NMR spectra were recorded on Bruker Avance III 500 spectrometer operated at 500 MHz. Spectra were calibrated relative to solvent´s residual proton chemical shift.

**List of Figures**

**Figure S1**. (a) size-distribution of 15 nm core AuNPs derived from TEM; (b) exemplary TEM image of 15 nm AuNPs; (c) size-distribution of 30 nm core AuNPs derived from TEM; (d) exemplary TEM image of 30 nm AuNPs.

**Figure S2.** (a) extinction spectra of 30 nm AuNPs and 30Au-4PEGSH nanohybrids in water; (b) DLS size-distribution curves of 30 nm AuNPs and 30Au-4PEGSH nanohybrids in water.

**Figure S3.** (a) Extinction spectra of 15Au-PEGSH and 15Au-PEGSH-CdSe and (b) 15Au-PSt-b-PEG and 15Au-PSt-b-PEG-CdSe used for the assembly yield quantification.

**Figure S4.** (a) size-distribution of satellite CdSe/CdS/ZnS QDs derived from TEM; (b) exemplary TEM image of 15Au-4PEGSH- CdSe/CdS/ZnS QDs nanoassemblies; (c) size-distribution of satellite AuNPs derived from TEM; (d) exemplary TEM image of 15Au-4PEGSH-Au nanoassemblies.

**Figure S5.** TEM image of (a) 30Au-PEGSH-CdSe/CdS/ZnS; (b) 30Au-PEGSH-InP/ZnSe/ZnS; (c) 30Au-4PEGSH-InP/ZnSe/ZnS nanoassembly.

**Figure S6.** (a) extinction of CdSe/CdS/ZnS QDs in NMF and 30Au-CdSe/CdS/ZnS core-satellite nanoassemblies in DMF; (b) emission spectra of CdSe/CdS/ZnS QDs in NMF and 30Au-CdSe/CdS/ZnS core-satellite nanoassemblies in DMF; (c) extinction of InP/ZnSe/ZnS QDs in NMF and 30Au-InP/ZnSe/ZnS core-satellite nanoassemblies in water; (d) emission spectra of InP/ZnSe/ZnS QDs in NMF and 30Au-InP/ZnSe/ZnS core-satellite nanoassemblies in water.

**Figure S7.** Synthetic scheme of PSt-*b*-PEG via RAFT polymerization and (a) GPC curves of PSt-*b*-PEG; (b) ^1^H NMR of PSt-*b*-PEG in CDCl_3_.

**Figure S8.** (a) TRPL spectra of 30Au-PEGSH-CdSe/CdS/ZnS, 30Au-PSt-*b*-PEG-CdSe/CdS/ZnS and CdSe/CdS/ZnS in different solvents and respective decay parameters at QD´s emission wavelength; (b) 30Au-PSt-*b*-PEG-CdSe nanoassembly in DMF (swollen polymer brush) and CHCl_3_ (extended polymer brush).

**Figure S1.** (a) size-distribution of 15 nm core AuNPs derived from TEM; (b) exemplary TEM image of 15 nm AuNPs; (c) size-distribution of 30 nm core AuNPs derived from TEM;
(d) exemplary TEM image of 30 nm AuNPs.

**
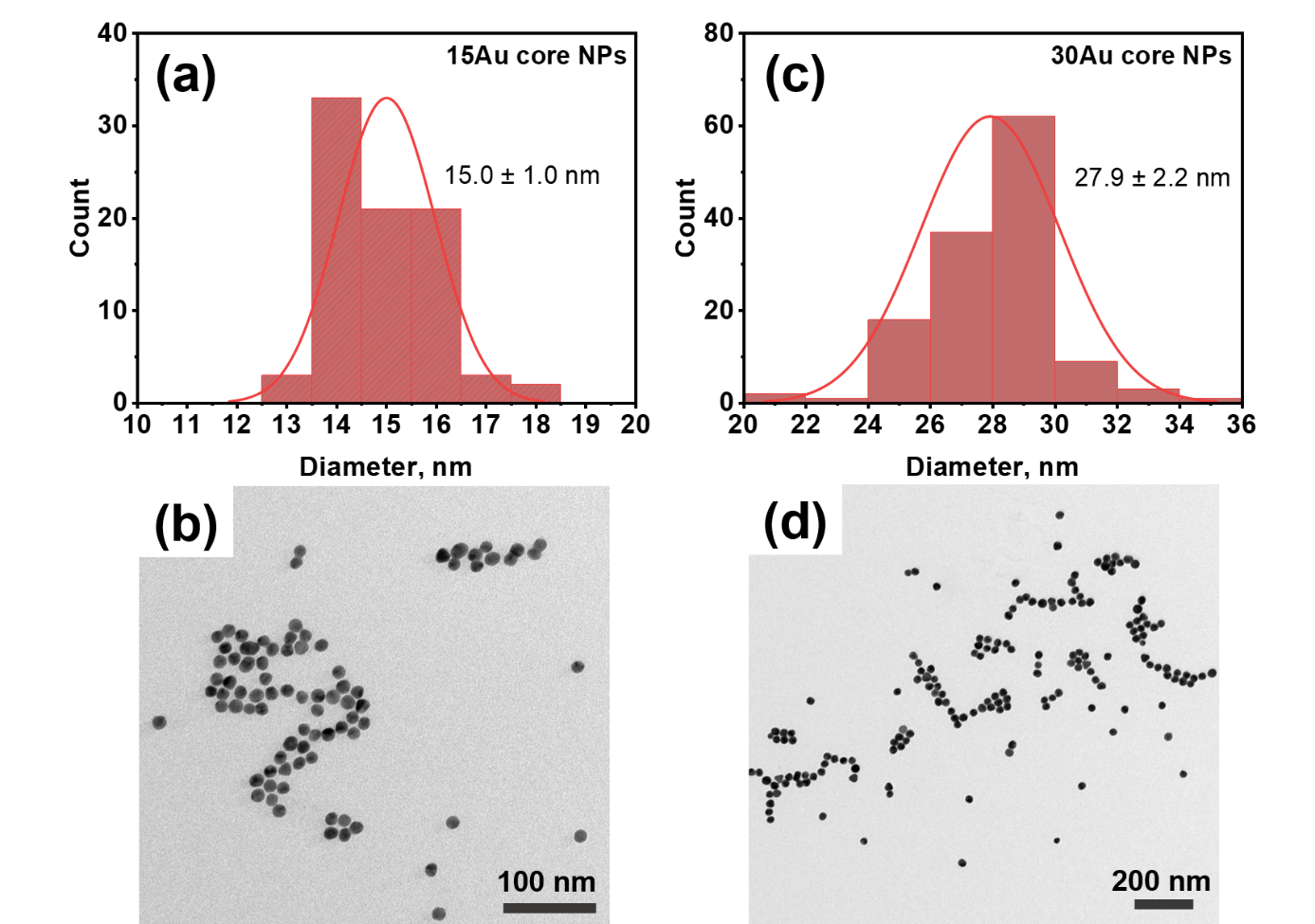
**

**Figure S2.** (a) extinction spectra of 30 nm AuNPs and 30Au-4PEGSH nanohybrids in water; (b) DLS size-distribution curves of 30 nm AuNPs and 30Au-4PEGSH nanohybrids in water.


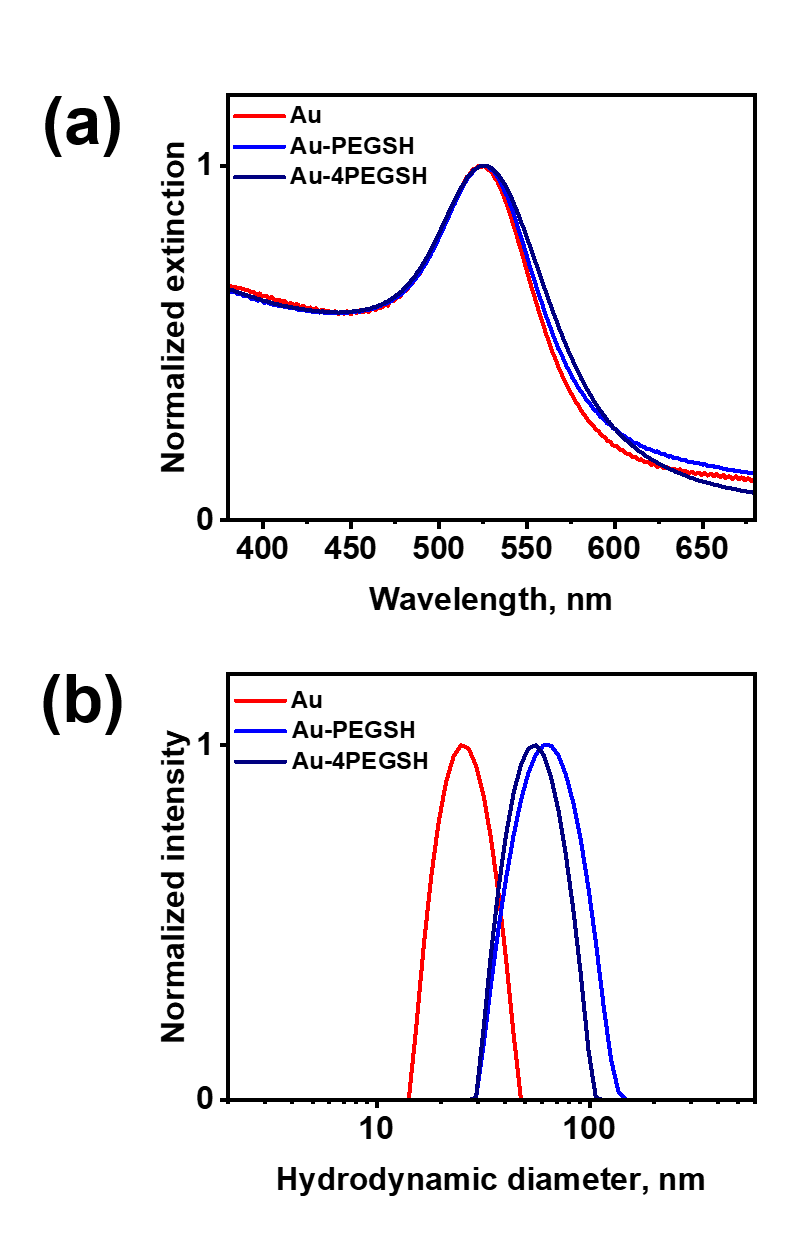


**Figure S3.** (a) Extinction spectra of 15Au-PEGSH and 15Au-PEGSH-CdSe and (b) 15Au-PSt-b-PEG and 15Au-PSt-b-PEG-CdSe used for the assembly yield quantification.


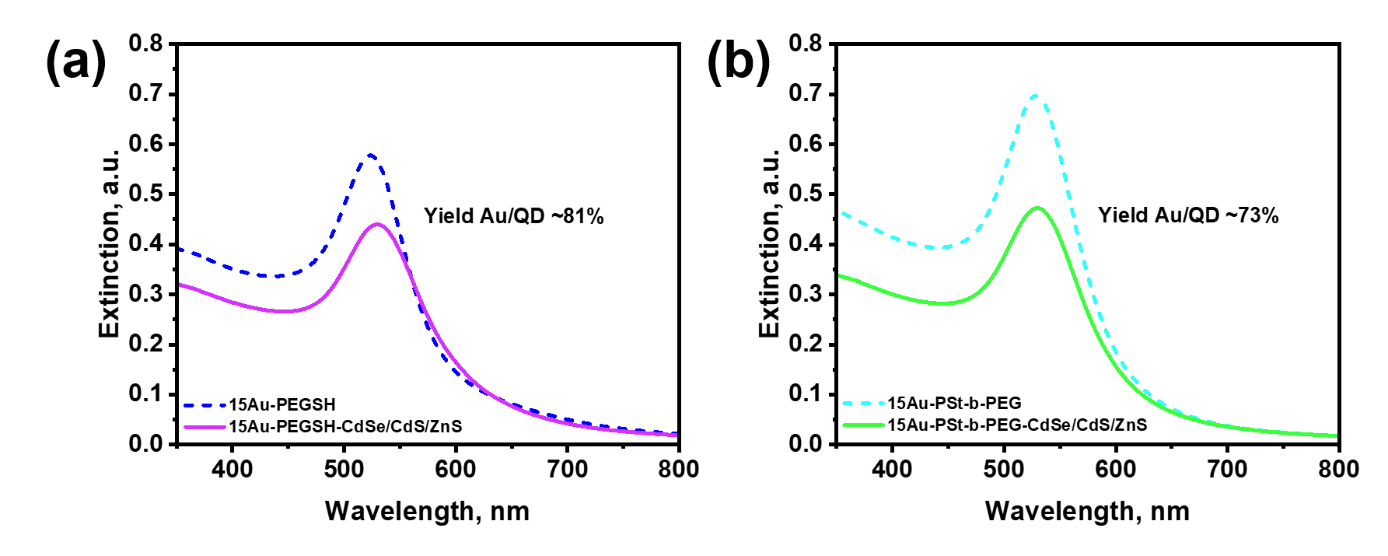


**Figure S4.**  (a) size-distribution of satellite CdSe/CdS/ZnS QDs derived from TEM; (b) exemplary TEM image of 15Au-4PEGSH- CdSe/CdS/ZnS QDs nanoassemblies; (c) size-distribution of satellite AuNPs derived from TEM; (d) exemplary TEM image of 15Au-4PEGSH-Au nanoassemblies.

**
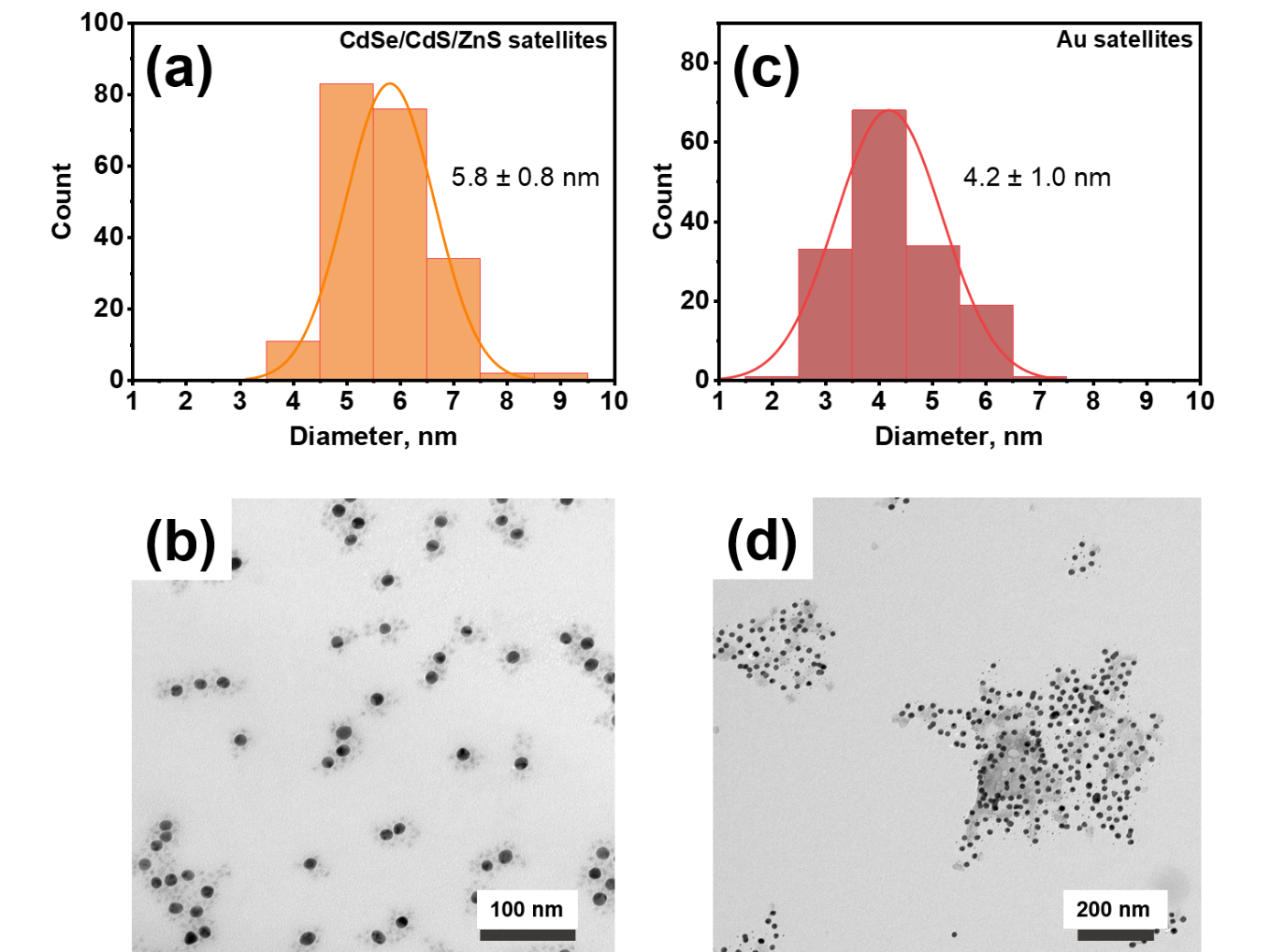
**

**Figure S5.** TEM image of (a) 30Au-PEGSH-CdSe/CdS/ZnS; (b) 30Au-PEGSH-InP/ZnSe/ZnS; (c) 30Au-4PEGSH-InP/ZnSe/ZnS nanoassembly.


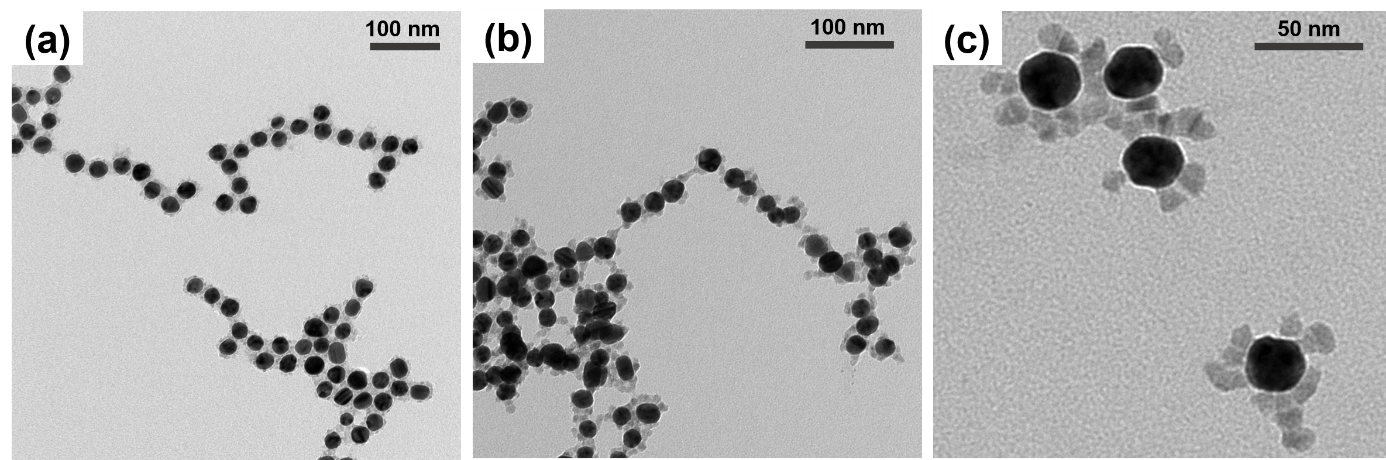


**Figure S6.**  (a) extinction of CdSe/CdS/ZnS QDs in NMF and 30Au-CdSe/CdS/ZnS core-satellite nanoassemblies in DMF; (b) emission spectra of CdSe/CdS/ZnS QDs in NMF and 30Au-CdSe/CdS/ZnS core-satellite nanoassemblies in DMF; (c) extinction of InP/ZnSe/ZnS QDs in NMF and 30Au-InP/ZnSe/ZnS core-satellite nanoassemblies in water; (d) emission spectra of InP/ZnSe/ZnS QDs in NMF and 30Au-InP/ZnSe/ZnS core-satellite nanoassemblies in water.


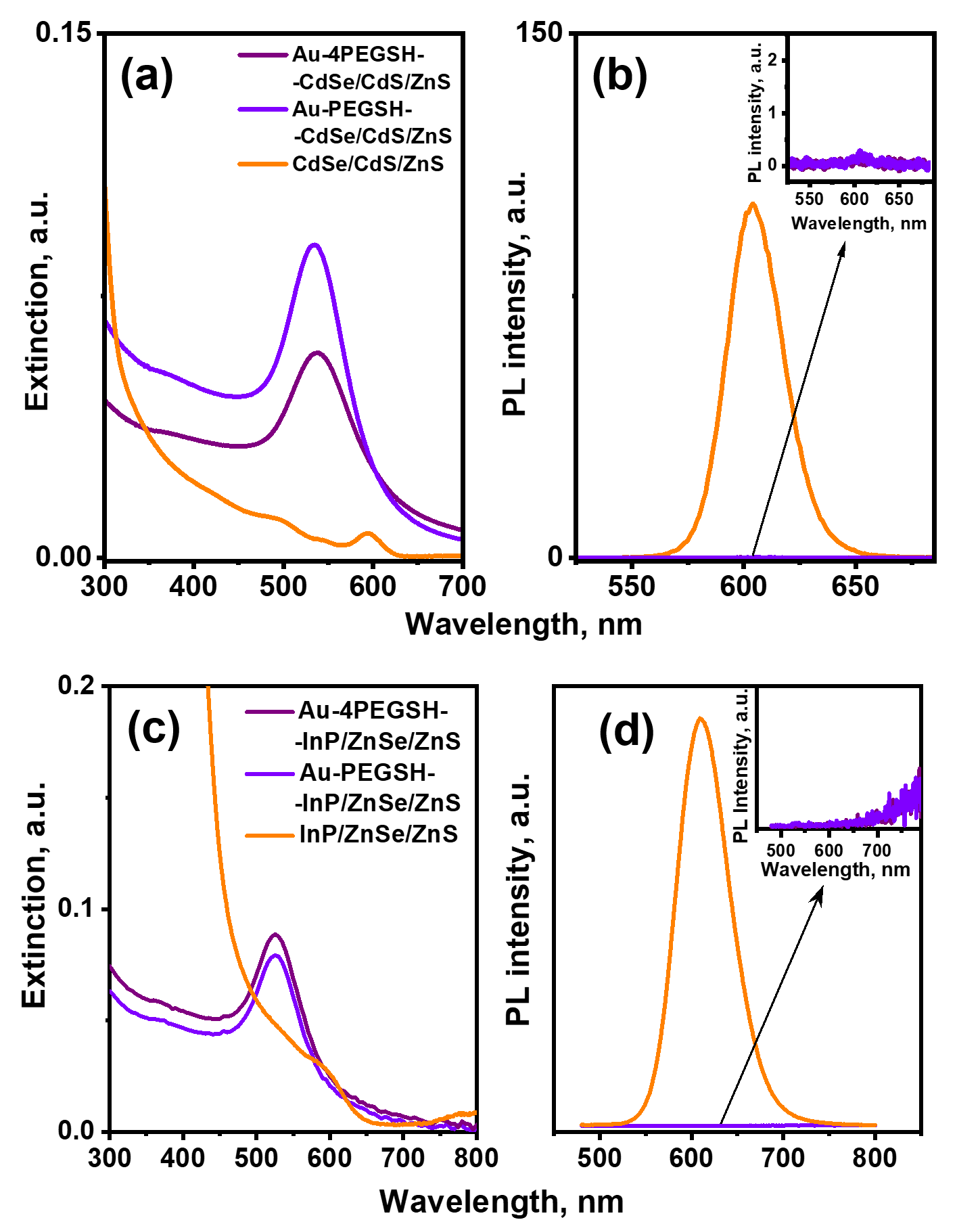


**Figure S7.** Synthetic scheme of PSt-*b*-PEG via RAFT polymerization and (a) GPC curves of PSt-*b*-PEG; (b) ^1^H NMR of PSt-*b*-PEG in CDCl_3_.


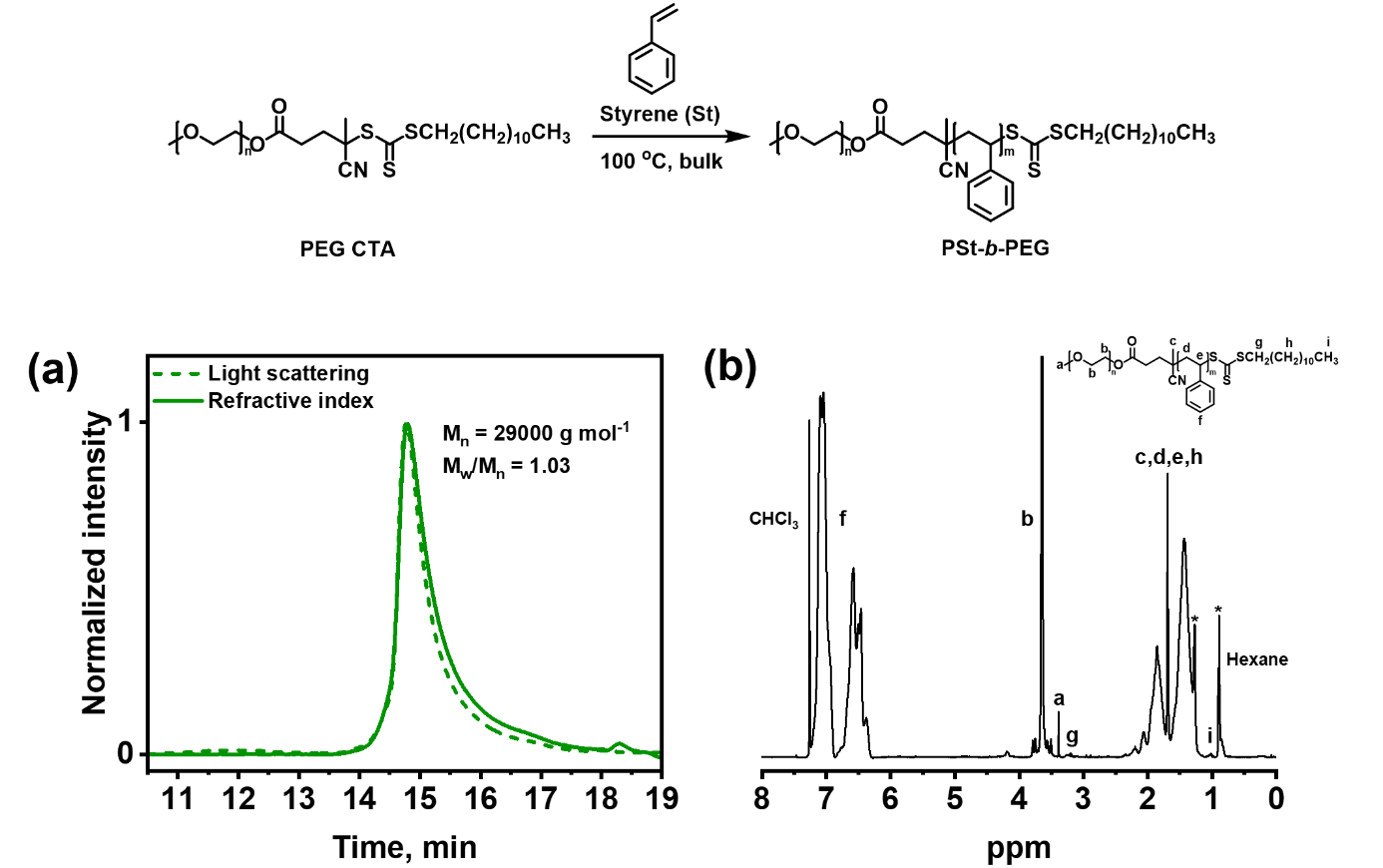


**Figure S8.** (a) TRPL spectra of 30Au-PEGSH-CdSe/CdS/ZnS, 30Au-PSt-*b*-PEG-CdSe/CdS/ZnS and CdSe/CdS/ZnS in different solvents and respective decay parameters at QD´s emission wavelength; (b) 30Au-PSt-*b*-PEG-CdSe nanoassembly in DMF (swollen polymer brush) and CHCl_3_ (extended polymer brush).


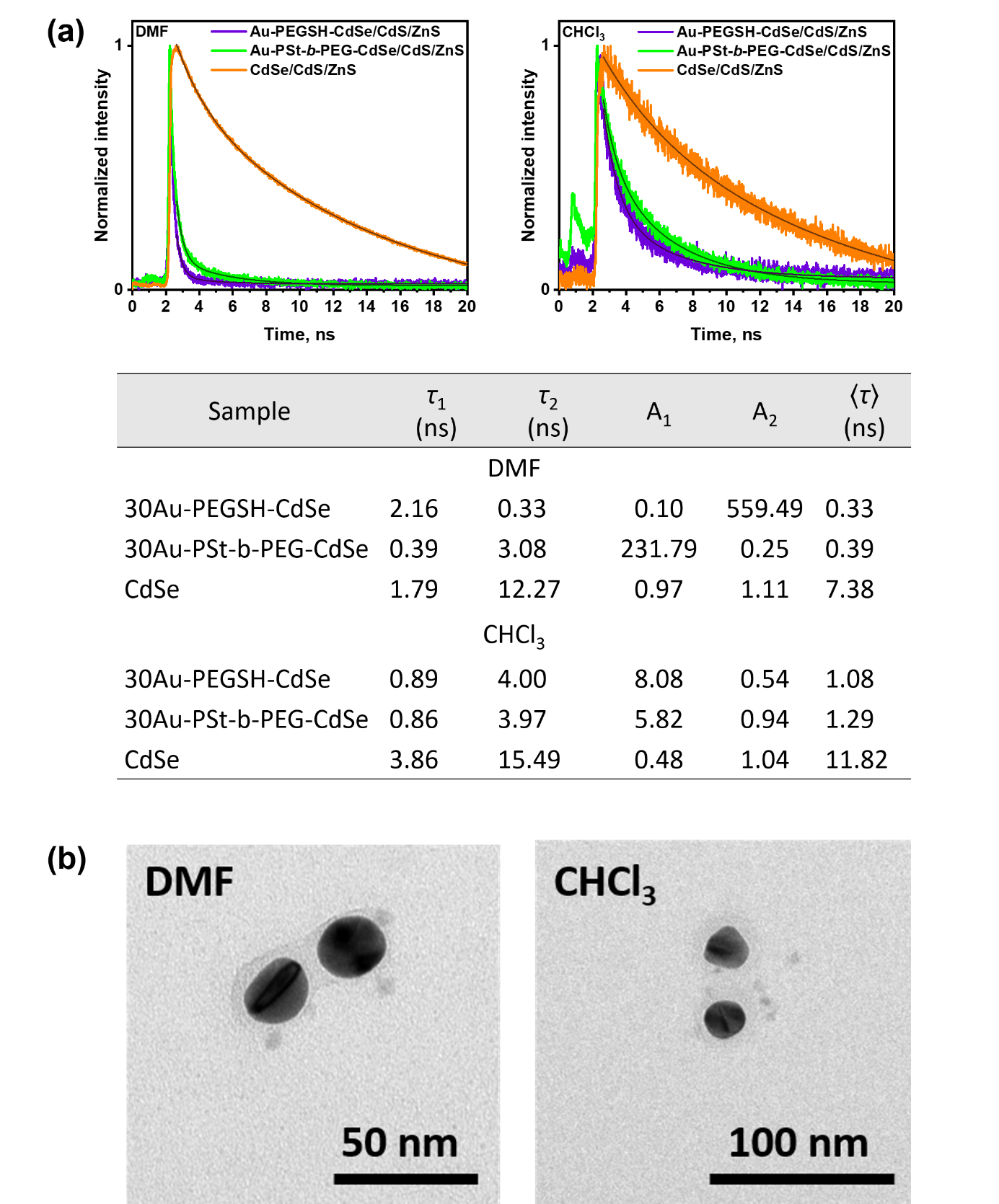


.

**References**

(S1) Bastús, N. G.; Comenge, J.; Puntes, V. Kinetically Controlled Seeded Growth Synthesis of Citrate-Stabilized Gold Nanoparticles of up to 200 Nm: Size Focusing versus Ostwald Ripening. *Langmuir* **2011**, *27* (17), 11098–11105.

(S2) Rossner, C.; Ebeling, B.; Vana, P. Spherical Gold-Nanoparticle Assemblies with Tunable Interparticle Distances Mediated by Multifunctional RAFT Polymers. *ACS Macro Lett.* **2013**, *2* (12), 1073–1076.

(S3) Van Avermaet, H.; Schiettecatte, P.; Hinz, S.; Giordano, L.; Ferrari, F.; Nayral, C.; Delpech, F.; Maultzsch, J.; Lange, H.; Hens, Z. Full-Spectrum InP-Based Quantum Dots with Near-Unity Photoluminescence Quantum Efficiency. *ACS Nano* **2022**, *16* (6), 9701–9712.

(S4) Saha, A.; Yadav, R.; Rivaux, C.; Aldakov, D.; Reiss, P. Water-Soluble Alumina-Coated Indium Phosphide Core–Shell Quantum Dots with Efficient Deep-Red Emission Beyond 700 Nm. *Small* **2024**, *20* (45), 2404426.

(S5) Rossner, C.; Vana, P. Planet-Satellite Nanostructures Made to Order by RAFT Star Polymers. *Angew. Chem. Int. Ed.* **2014**, *53* (46), 12639–12642.

(S6) Lu, J.; Xue, Y.; Shi, R.; Kang, J.; Zhao, C.-Y.; Zhang, N.-N.; Wang, C.-Y.; Lu, Z.-Y.; Liu, K. “A non-sacrificial method for the quantification of poly(ethylene glycol) grafting density on gold nanoparticles for applications in nanomedicine” *Chem. Sci.* **2019,** *10,* 2067.

(S7) Scarabelli, L.; Sánchez-Iglesias, A.; Pérez-Juste, J.; Liz-Marzán, L. M. A “Tips and Tricks” Practical Guide to the Synthesis of Gold Nanorods. *J. Phys. Chem. Lett.* **2015**, *6* (21), 4270–4279.

(S8) Haiss, W.; Thanh, N. T. K.; Aveyard, J.; Fernig, D. G. “Determination of Size and Concentration of Gold Nanoparticles from UV−Vis Spectra” *Anal. Chem.* **2007,** *79* (11), 4215–4221.

(S9) Yu, W. W.; Qu, L.; Guo, W.; Peng, X. “Experimental Determination of the Extinction Coefficient of CdTe, CdSe, and CdS Nanocrystals” *Chem. Mater.* **2003,** *15* (14), 2854–2860.
